# The expression and clinical significance of PLK1/p-PLK1 protein in NK/T cell Lymphoma

**DOI:** 10.1186/s13000-023-01413-w

**Published:** 2023-11-30

**Authors:** Zhiqi Zhang, Enjie Liu, Dandan Zhang, Wugan Zhao, Guannan Wang, Yanping Zhang, Yajun Huo, Chongli Zhang, Wencai Li

**Affiliations:** Department of Pathology, The First Affiliated Hospital of Zhengzhou University, Zhengzhou, 450052 Henan China

**Keywords:** NK/T cell Lymphoma, Polo-like kinase 1 protein (PLK1), p-PLK1

## Abstract

**Aims:**

To investigate the expression of polo-like kinase 1 protein (PLK1) and its phosphorylation level (p-PLK1) in extranodal NK/T cell lymphoma (NKTCL) and their correlation with clinical characteristics and prognosis.

**Methods:**

We collected 40 cases of NKTCL (referred to as the experimental group), which received diagnoses at the First Affiliated Hospital of Zhengzhou University between January 2018 and October 2022. Concurrently, we assembled a control group, including 20 cases afflicted with nasopharyngeal mucosal lymphoid hyperplasia diseases during the same timeframe. We utilized immunohistochemical techniques to evaluate the levels of PLK1 and p-PLK1 expression in both the experimental and control groups. Subsequently, we conducted an analysis to identify disparities in their expression and explore their relationships with clinical characteristics and patient prognosis.

**Results:**

Among the 40 NKTCL patients, there were 27 males and 11 females, with a median age of 51 years (range 12–80 years). Compared to the control group, the tissue samples of NKTCL patients exhibited significantly elevated expression levels and active phosphorylation levels of PLK1 (*P* < 0.05). Correlation analysis of the immunohistochemical H score and Ki-67 positive rate of PLK1 and p-PLK1, revealed a significant positive correlation for both (*P* < 0.0001, each). No statistically significant differences were observed in the distribution of PLK1 and p-PLK1 expression in NKTCL patients with respect to gender, age, Ann Arbor stage, PINK-E score, B-symptoms, lactate dehydrogenase, β2-microglobulin, blood EBV-DNA, bone marrow invasion, and lymph node metastasis (*p* > 0.05). Grouping based on PLK1 and p-PLK1 immunohistochemical H-scores revealed that the high expression of PLK1 and p-PLK1 was associated with poor prognosis.

**Conclusions:**

The expression levels and active phosphorylation levels of PLK1 were significantly increased in NK/T cell lymphoma, and patients with overexpression of PLK1 and p-PLK1 had a poorer prognosis.

## Introduction

NK/T cell lymphoma (NKTCL) is a common malignant tumor with high invasion and poor prognosis in China. It accounts for about 6.4% of non-Hodgkin lymphoma in China and is highly associated with Epstein-Barr virus (EBV) infection [1]. Most NKTCL cases (80–90%) are initially diagnosed with extranodal involvement, mainly in the nasal cavity and adjacent areas [2]. The treatment methods for NKTCL include radiotherapy, chemotherapy, immunotherapy, etc [3]; NKTCL patients, especially those in terminal and advanced stages, still have poor prognosis [4]. In recent years, progress has been made in the pathogenesis and clinical treatment of NKTCL [5], the treatment regimens containing asparaginase have better effects on NKTCL, such as the SMILE regimen, AspaMetDex regimen, IMEP-L-asp regimen, P-GEMOX regimen, and DDGP regimen [6]. The CR rate of the above scheme can reach between 45% and 67.5%, and the therapeutic effect is very significant. Even so, nearly 30% of patients still do not achieve complete remission with the current chemotherapy regimen. Therefore, effective treatment exploration is still urgently needed for NKTCL.

Polo-like kinase 1 (PLK1) is a highly conserved Serine/Threonine protein kinase and an important member of the Polo-like kinase family. Through phosphorylation of different specific substrates, PLK1 plays an important role in the cell cycle process and multiple stages of mitosis and is a key protein to promote cell proliferation. The expression of PLK1 is up-regulated in breast cancer, melanoma, non-small cell lung cancer, colorectal cancer, prostate cancer, pancreatic cancer, ovarian cancer, and other types of tumors. PLK1 regulates its function by interacting with tumor-related proteins, affecting malignant biological behaviors such as tumor proliferation, invasion, and migration, and is associated with poor prognosis of the patients [7, 8]. Multiple studies have found that PLK1 also plays an important role in lymphopoietic and immune-related tumors [9]. Research has shown that inhibiting PLK1 can promote the reactivation of latent infection state EBV and promote tumor cell death caused by EBV infection [10]. Preliminary research has also shown that the expression level of PLK1 in NKTCL is increased, while Resveratrol and icariin could inhibit the activity of PLK1 and promote the apoptosis of NKTCL tumor cells [2, 11], which suggests that PLK1 may play an important role in the occurrence and development of NKTCL. It has been proved that PLK1 is activated in the G2 phase by phosphorylation of T210 residues in its T-ring before entering Mitosis [12], although the abnormal activation of PLK1 plays an important role in the occurrence and development of various lymphohematopoietic and immune-related tumors, the expression and mechanism of PLK1 and its phosphorylation levels in NKTCL, as well as their relationship with clinical pathological characteristics and prognosis of NKTCL, have not been reported yet.

## Materials and methods

### Clinical data

40 paraffin tissue samples of NKTCL patients diagnosed in the First Affiliated Hospital of Zhengzhou University according to the fourth revision “2017 WHO Classification of Lymphoid and Hematopoietic Tissue Tumors” were collected as the experimental group from January 2018 to October 2022. Collect the following clinical data through the hospital’s electronic medical record: age, gender, Ann Arbor stage, PINK-E score, presence or absence of B symptoms, Lactate dehydrogenase (LDH), β2 microglobulin, blood EBV-DNA, Ki-67 proliferation index, presence or absence of bone marrow invasion and lymph node metastasis, etc. In addition, 20 nasopharyngeal mucosa Lymphoid hyperplasia disease tissue samples were collected as the control group. All 40 cases of NKTCL and 20 cases of nasopharyngeal mucosa Lymphoid hyperplasia diseases were included in the study of expression differences. Two NKTCL patients who were lost in follow-up were excluded during survival analysis.

### Pathological examination

The tissue samples included in the study were re-examined (including routine HE staining and immunohistochemical staining) by two pathologists according to the fourth revision”2017 WHO Classification of Lymphoid and Hematopoietic Tissue Tumors》and confirmed.

### Immunohistochemical staining

Cut paraffin specimen into 3 μm thin slices, baked at 75℃ for 120 min, followed by immunohistochemical EnVision two-step method [13]. PLK1 (208G4) Rabbit mAb was purchased from Cell Signaling Technology with a dilution concentration of 1:100; Anti PLK1 (photo T210) antibody [EPNCIR167] was purchased from Abcam company with a dilution concentration of 1:150.

### Result interpretation

Observe HE staining sections under an optical microscope, and determine the distribution of tumor cells, the semi-quantitative H-score method [14] was used to score the staining of PLK1 and p-PLK1 in 40 cases of the experimental group and 20 cases of the control group. PLK1 and p-PLK1 positivity are localized in the nucleus. Firstly, determine the dyeing intensity: 0 (no staining), 1+(dim), 2+(medium), and 3+(strong). Secondly, calculate the percentage of cells (% Cells) at each staining intensity level. Finally, use the formula H-score = Σ(PI×I) = (percentage of cells of weak intensity × 1) + (percentage of cells of moderate intensity × 2) + (percentage of cells of strong intensity × 3) to calculate the H-score (Table 1). This process is reviewed and agreed upon by two pathologists, with scores higher than 1.5 indicating high expression and scores lower than 1.5 indicating low expression [15].

**Table 1:** H-score scoring criteria

staining intensity	score
no staining	0
dim	1+
medium	2+
strong	3+
Scoring indicators = Σ (Dyeing intensity score × Cell percentage at each staining intensity)	
A score of 1.5 is used as the limit, with higher values indicating high expression and lower values indicating low expression.	

### Follow-up

The start time is the date of diagnosis, and the deadline is February 15, 2023. Clinical outcome: Overall survival (OS) refers to the time from diagnosis to the occurrence of death from any cause or the end of follow-up.

### Statistical analysis

SPSS 26.0 statistical software and GraphPad Prism 8.0 were used for data analysis and plotting. The Chi-squared test or Fisher’s exact test was used to compare the different parameters of studies. The Spearman rank correlation method was used for correlation analysis. The OS and generation of survival curves were performed using the Kaplan-Meier method, and significance was determined using the log-rank test. A value of *p* < 0.05 was considered a statistically significant difference.

## Results

### Clinical characteristics (table 2)

Among the 40 patients, there were 27 males and 13 females, ranging in age from 12 to 80 years old (median age 51 years old). The follow-up time was 0.5–48 months, with a median follow-up time of 24 months. As of the follow-up endpoint, 11 patients died, 2 patients were lost to follow-up, and the remaining 27 patients have survived to this day.

**Table 2:** The relationship between the expression of PLK1 and p-PLK1 and clinical characteristics in 40 cases of NK/T-cell lymphoma [cases (%)]

Clinical features	Cases	Low expression of PLK1	High expression of PLK1	*P* value	Low expression of p-PLK1	High expression of p-PLK1	*P* value
Gender				0.053			0.145
Male	27	11(40.7)	16(59.3)		11(40.7)	16(59.3)	
Female	13	9(69.2)	4(30.8)		9(69.2)	4(30.8)	
Age(years)				0.204			0.204
≤ 51	20	12(60.0)	8(40.0)		12(60.0)	8(40.0)	
> 51	20	8(40.0)	12(60.0)		8(40.0)	12(60.0)	
Ann Arbor stage				0.677			0.096
I/II stage	28	14(50.0)	14(50.0)		17(60.7)	11(39.3)	
III/IV stage	12	6(50.0)	6(50.0)		3(25.0)	9(75.0)	
PINK-E score				0.781			0.781
0–1 score	28	13(46.4)	15(53.6)		14(50.0)	14(50.0)	
2–3 score	12	7(58.3)	5(41.7)		6(50.0)	6(50.0)	
B symptoms				0.775			0.726
yes	19	9(47.4)	10(52.6)		9(47.4)	10(52.6)	
no	21	11(52.4)	10(47.6)		11(52.4)	10(47.6)	
LDH(U/L)				0.97			0.769
< 245	23	12(52.2)	11(47.8)		12(52.2)	11(47.8)	
≥ 245	14	7(50.0)	7(50.0)		7(50.0)	7(50.0)	
β2 microglobulin(mg/L)				0.248			0.452
< 3	27	14(51.9)	13(48.1)		15(55.6)	12(44.4)	
≥ 3	8	3(37.5)	5(62.5)		3(37.5)	5(62.5)	
Blood EBV-DNA				0.274			0.346
Normal	21	12(57.1)	9(42.9)		13(62.0)	8(38.0)	
Increase	5	2(40.0)	3(60.0)		3(60.0)	2(40.0)	
Bone marrow invasion				0.455			0.576
Yes	1	0(00.0)	1(100.0)		0(00.0)	1(100.0)	
No	32	16(50.0)	16(50.0)		16(50.0)	16(50.0)	
Lymph node metastasis				0.591			0.635
Yes	3	2(66.7)	1(33.3)		1(33.3)	2(66.7)	
No	31	15(48.4)	16(51.6)		15(48.4)	16(51.6)	

### Expression and comparison of PLK1 and p-PLK1 in the experimental and control groups

**Fig. 1:**
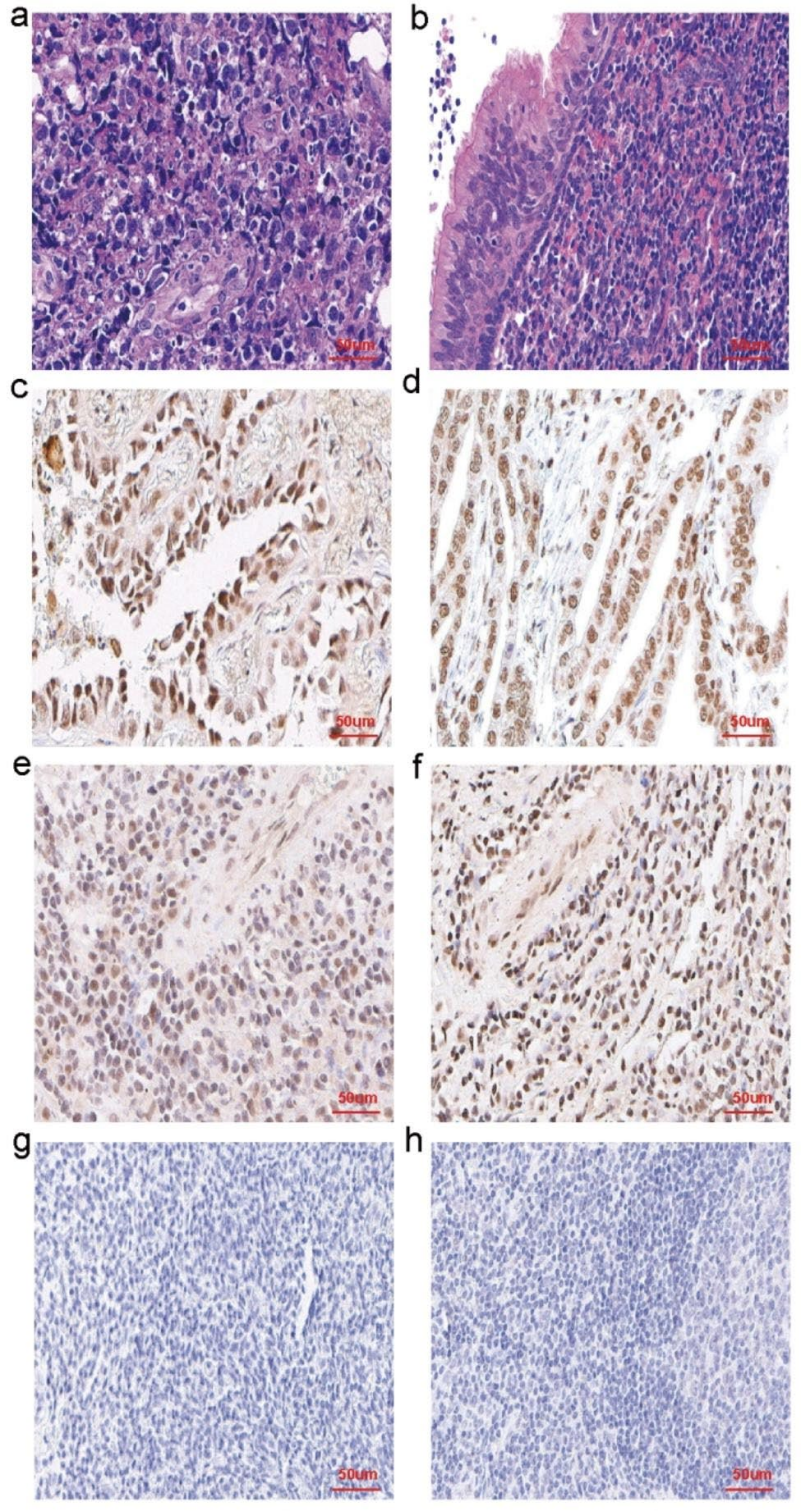
**a** NKTCL tissue showed diffuse infiltration of tumor cells, destruction of blood vessels, medium to large cells, irregular nuclei, and granular chromatin **b** Nasopharyngeal mucosa lymphoid hyperplasia diseases tissue showed normal nasal mucosa with lymphoid hyperplasia **c** PLK1 positive control showed positive expression in the nucleus of lung cancer **d** p-PLK1 positive control showed positive expression in the nucleus of gastric cancer cells **e** Expression of PLK1 showed positive in NKTCL tissue **f** Expression of p-PLK1 showed positive in NKTCL tissue **g** Expression of p-PLK1 showed negative in Nasopharyngeal mucosa lymphoid hyperplasia diseases tissue **h** Expression of p-PLK1 showed negative in Nasopharyngeal mucosa lymphoid hyperplasia diseases tissue

Positive signals for PLK1 and p-PLK1 were localized in the nucleus, with 100% positive expression in tumor cells among the 40 NKTCL patients. The proportion of high expression was 45% (18/40) for PLK1 and 55% (22/40) for p-PLK1, respectively. The staining intensity of p-PLK1 was stronger than that of PLK1. In contrast, the control group, exhibited no expression or weak expression of both PLK1 and p-PLK1. The expression and staining of both proteins in NKTCL patients were higher compared to the control group (Fig. 1).

### Correlation between the expression of PLK1, p-PLK1, and Ki-67 in NKTCL tissue

**Fig. 2:**
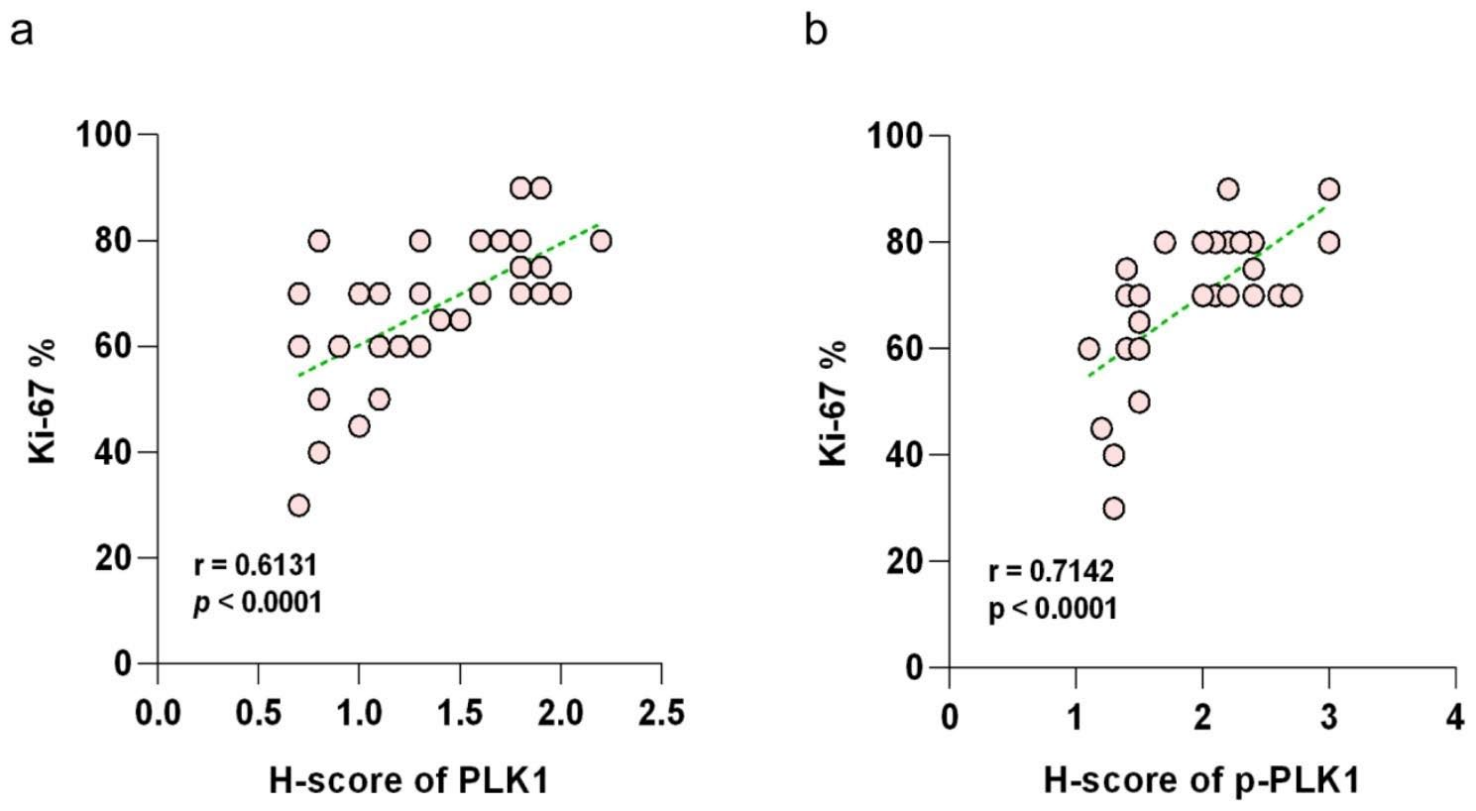
Expression level of PLK1 is positively correlated with the Ki-67 positive rate (**2a**). The expression level of p-PLK1 is positively correlated with the Ki-67 positive rate (**2b**)

There is a strong consistency in the expression of PLK1 and p-PLK1 in the NKTCL tissue. Among the 40 cases of NKTCL tissue, 17 cases showed low expression of both PLK1 and p-PLK1, 17 cases exhibited high expression of both, 5 cases had low PLK1 expression but high p-PLK1 expression, and 1 case had high PLK1 expression but low p-PLK1 expression. Spearman’s correlation analysis indicated a moderate positive correlation between the expression of these two proteins in NKTCL (r = 0.498, *P* = 0.0011). Furthermore, a strong positive correlation was observed between the expression level of PLK1 and the Ki-67 positive rate (r = 0.613, *P* < 0.0001) (Fig. 2a), and a similarly strong positive correlation was found between the expression level of p-PLK1 and the Ki-67 positive rate(r = 0.714, *P* < 0.0001) (Fig. 2b).

### Relationship between the expression of PLK1 and p-PLK1 and the clinical characteristics of NKTCL patients (table 2)

Statistical analysis was conducted on the clinical data of 40 patients, revealing no statistically significant differences in the distribution of PLK1 and p-PLK1 expression among NKTCL patients with regard to gender, age, Ann Arbor stage, PINK-E score, B-symptoms, lactate dehydrogenase, β2-microglobulin, blood EBV-DNA, bone marrow invasion, and lymph node metastasis (*p* > 0.05).

### Survival analysis

**Fig. 3:**
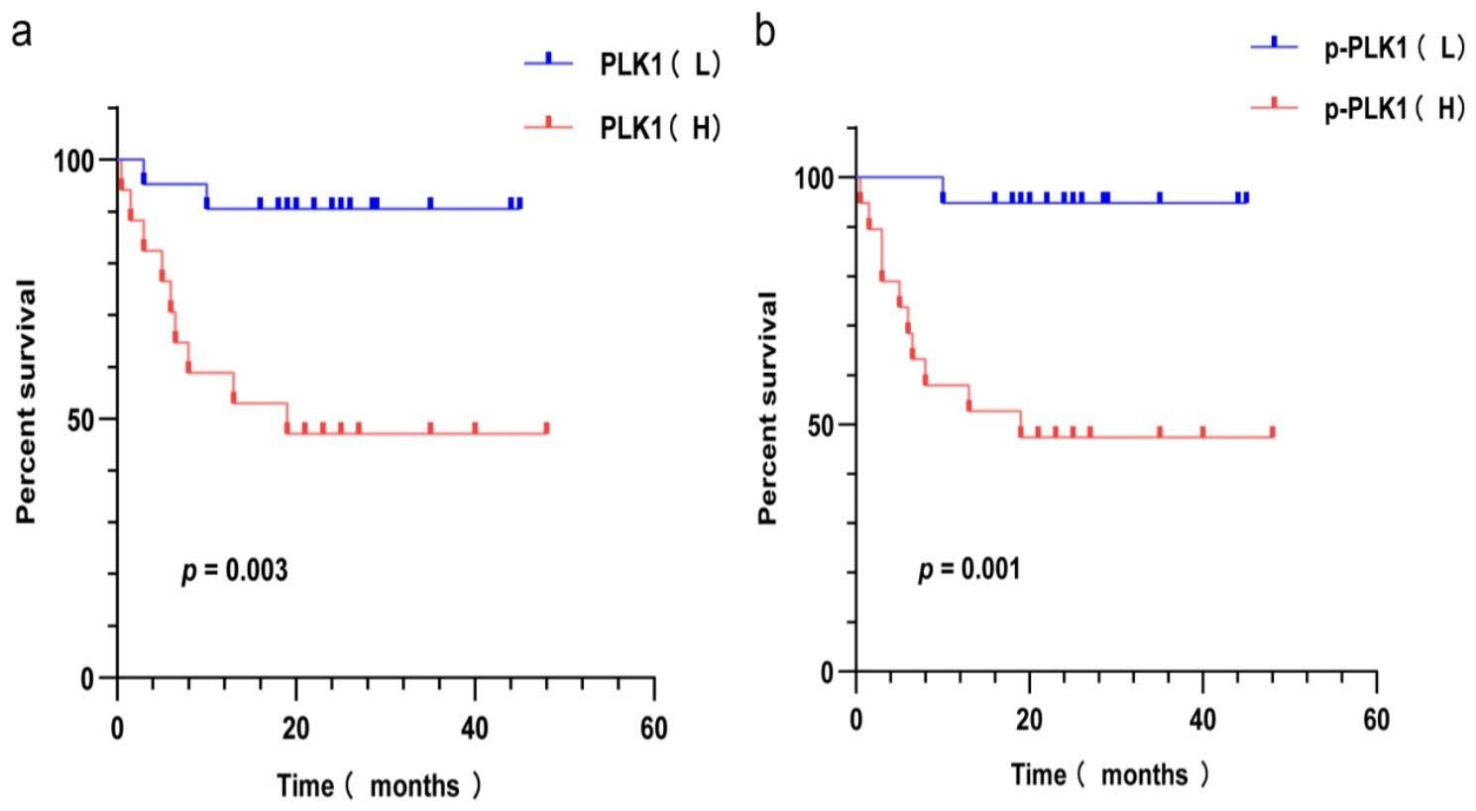
The relationship between low and high expression of PLK1 and the overall survival rate of NKTCL patients (**3a**). The relationship between low and high expression of p-PLK1 and overall survival in NKTCL patients (**3b**)

As of February 15, 2023, the median follow-up time was 24 months (range 1.5–48). Among the 38 patients who underwent treatment and follow-up, 11 passed away, resulting in a 5-year OS of 71.1% (27/38). Kaplan-Meier survival analysis revealed that the high expression group of PLK1 and p-PLK1 had significantly reduced 5-year OS compared to the low expression group. This difference was statistically significant according to the Log-rank (Mantel-Cox) test (χ²=8.649, *p =* 0.003 for Fig. 3a; χ² = 10.49, *p =* 0.001 for Fig. 3b).

### ROC curve analysis of PLK1 and p-PLK1 for prognosis judgment of NKTCL

**Fig. 4:**
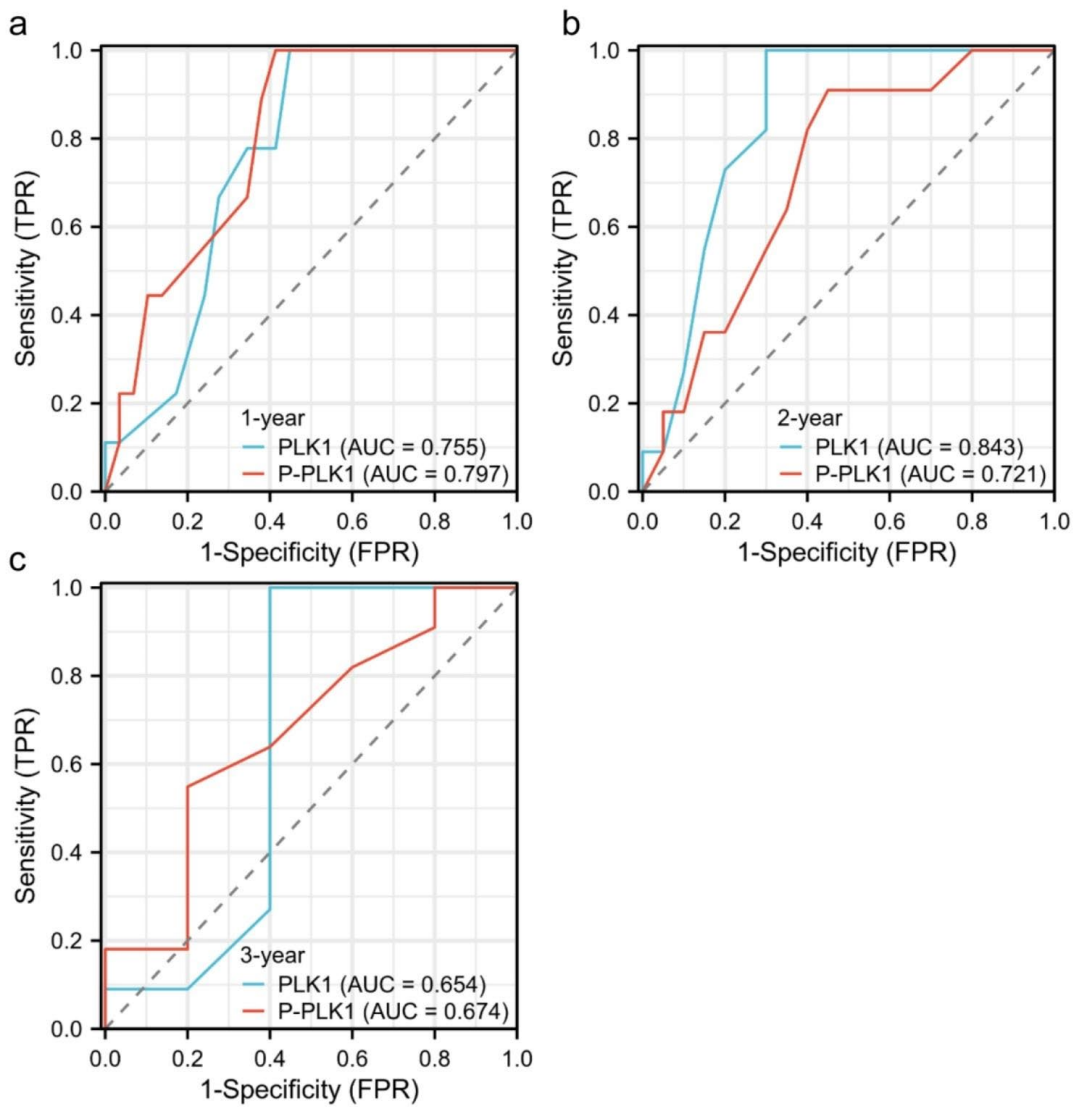
ROC curve analysis of PLK1 and p-PLK1 for prognosis judgment of NKTCL

For patients diagnosed with NKTCL for one year and three years, the AUC of p-PLK1 for predicting NKTCL prognosis was 0.797 and 0.674, respectively, slightly higher than that of PLK1, which had AUC values of 0.755 and 0.654. However, for patients diagnosed with NKTCL for 2 years, the AUC value of PLK1 for predicting their prognosis was 0.843, surpassing the 0.721 AUC value of p-PLK1 (Fig. 4).

## Discussion

NK/T-cell lymphoma is a rare and highly aggressive subtype of non-Hodgkin lymphoma (NHL) with a poor prognosis. It is highly associated with EB virus infection, most commonly occurring in the nose and primarily involving NK cell or T-cell proliferation [16]. Its characteristics include damage and destruction of blood vessels, noticeable necrosis, and a cytotoxic immune phenotype [17]. The tumor cells exhibit a wide range of cytological variations, from small to large and anaplastic cells. Immunophenotypically, the malignant cells are positive for cCD3 (or CD2), CD56, cytotoxic markers (such as TIA-1, Granzyme B, and perforin), and EBV [5]. Sanjay de Mel and colleagues published a review summarizing the key molecular and pathogenic pathways in NKTCL. Among the numerous pathogenesis mechanisms they mentioned, DNA damage response struck me as a potential association between NKTCL and PLK1. A disrupted DNA damage response resulting from aberrations in ataxia telangiectasia-related (ATR) kinases can lead to significant genomic instability and may contribute to the chemoresistance of ENKTL [5]. Several regulatory factors closely related to the DDR pathway, including PLK1, are essential cell cycle regulators. When DNA damage occurs, PLK1 is inhibited by activated ATM, leading to cell cycle arrests at multiple points in mitosis [18]. The suppression of PLK1 can induce DDR and cell apoptosis in cancer cell lines. Thus, PLK1 is a promising target for cancer therapy [2]. The overexpression of PLK1 is linked to the formation, metastasis, prognosis and overall survival rate of various tumor types, including breast cancer, non-small cell lung cancer, prostate cancer, and non-Hodgkin lymphoma. Therefore, the expression of PLK1 is employed as a prognostic marker [19]. Interestingly, one study provided in vitro evidence that resveratrol, a natural nontoxic pleiotropic agent abundant in grapes, blueberries, and peanuts, exhibited anti-tumor effects by activating the DDR pathway in an ATM/Chk2/p53-dependent manner in ENKTL cell lines. Inhibition of PLK1 may be the key mechanism of REV in activating DDR [2]. Another study also discovered that icaritin, a monomer derived from icariin, induced proliferation inhibition and apoptosis of NKTCL both in vitro and in vivo. They identified the Polo-like kinase 1 (PLK1) gene and DNA damage response (DDR) as the targets of icaritin [11].

Hence, we hypothesized that PLK1 overexpression resulted in a DDR defect, which could be a crucial link in the pathogenesis of NKTCL. Further research is necessary to conform whether the specific results align with our hypothesis. This study initially assessed the expression of PLK1 and phosphorylated PLK1 proteins in NKTCL and explored their correlation with the clinical pathological characteristics and prognosis of NKTCL patients. The aim was to investigate the relationship between the expression of PLK1 and phosphorylated PLK1 proteins in NKTCL, clinical characteristics and prognosis, providing a theoretical basis for further studying the role of PLK1 and its active phosphorylation level in the pathogenesis of NKTCL. It is worth noting that PLK1 inhibitors have shown promising therapeutic effects in triple negative breast cancer and ovarian cancer patients [20]. Our findings offer preliminary evidence for the potential application of PLK1 inhibitors in NKTCL, and further research could benefit more patients.

The general clinical characteristics of 40 NKTCL patients are largely consistent with those reported in the literature, predominantly occurring in males and in adults. Among them, one case is a minor, aged 12–80 years, with a median age of 51 years. The median age of NKTCL reported in the literature ranges from 46 to 60 years old, with ages spanning from 9 to 89 years old. Cases of NKTCL in children and adolescents are rare [21, 22]. Ann Arbor staging is mainly in stages I to II, with 32 cases occurring in the nasopharynx, accounting for approximately 80% of all cases. Eight cases occur in non-nasopharyngeal NKTCL, primarily in the ileum, skin, testicles, etc. [5]. It is reported that NKTCL often arises in the nasal cavity, while extranasal involvement is common in the skin, gastrointestinal tract, testicles, and other sites. Some cases exhibit systemic symptoms such as fever, night sweats, and weight loss. Bone marrow invasion and lymph node metastasis are rare. In this study, five cases had serum EBV-DNA levels higher than normal, and all cases were positive for EBER in situ hybridization. While the exact mechanism remains unclear, EBV appears to play a crucial role in the pathogenesis of NKTCL, affecting patients of all races [23]. Therefore, EBER in situ hybridization is also necessary for the pathological diagnosis of NKTCL [24], and measuring circulating EBV-DNA levels in the blood as a highly sensitive tumor marker holds significant has great practical value for post-treatment monitoring [25].

Ki-67 serves as an indicator for assessing the extent of cellular proliferation. It becomes evident during the phases of cell division within the cell cycle (G1, S, G2 and Mitosis), but remains absent during the intercellular phase. Ki-67 finds utility in appraising the proliferative vigor of neoplastic cells, a parameter intimately linked to cellular proliferation and the unfavorable clinical progression of diverse neoplasms. The manifestation of the Ki-67 index exhibits variability within NK/T-cell lymphoma. It was previously elucidated by Pongpruttipan et al. [26] that Ki-67 > 40% stands as an autonomous determinant significantly impacting the prognosis of NK/T-cell lymphoma. Studies have corroborated that the overall survival rate in extranodal NK/T-cell lymphoma featuring elevated Ki-67 expression is diminished [27]. PLK1, emerges as a pivotal controller of eukaryote cellular division. Its customary presence manifests in cells engaged in mitotic proliferation, but it becomes overexpressed across multiple forms of human malignancies. In this investigation, Spearman correlation analysis was initiated to ascertain the connection between PLK1 and p-PLK1 expression and the Ki-67 proliferation index among 40 cases of NK/T-cell lymphoma. The findings distinctly demonstrated a robust positive correlation between the levels of PLK1, p-PLK1 and the Ki-67 positivity rate (r = 0.613, r = 0.714, both *P* < 0.0001). Upon delving into the interplay between PLK1, p-PLK1 and the Ki-67 proliferation index, it was discerned that individuals with elevated expression of both PLK1 and p-PLK1 proteins, along with a high Ki-67 proliferation index, exhibited a bleak clinical prognosis. This constellation of factors may potentially serve as an indicator for prognostic evaluation in NK/T-cell lymphoma.

Within this investigation, the immunohistochemical staining of PLK1 and p-PLK1 was conducted in 40 cases of NKTCL patients, and juxtaposed with 20 cases of nasal mucosal inflammatory tissue. The outcomes unequivocally delineated a substantial upsurge in the expression of PLK1 and p-PLK1 within NKTCL as compared to the control group. Furthermore, a robust positive correlation between PLK1 and p-PLK1 expression was evident (r = 0.6, *P* < 0.01). In analyzing the experimental results, we observed that the phosphorylation level of PLK1 protein activity increased in comparison to the expression level of total PLK1 protein, suggesting that PLK1 protein is activated in NKTCL. The mechanism of this activation requires further research.

The Kaplan-Meier survival analysis revealed that in comparison to the low expression cohort, the OS in the high-expression group of PLK1 and its active phosphorylation levels exhibited a noteworthy decrease. As indicated in prior research, PLK1 exhibits an association with unfavorable prognosis of patients in breast cancer [28], hepatocellular carcinoma [29], colorectal cancer, synovial sarcoma, lung adenocarcinoma and various other malignancies.

The Chi-squared test was employed for the examination of the association between PLK1 expression, phosphorylation level, and diverse clinical attributes. The outcomes revealed no statistically significant distinctions in the distribution of PLK1 expression and p-PLK1 among NKTCL patients concerning gender, age, Ann Arbor stage, PINK-E score, B-symptoms, lactate dehydrogenase, β2-microglobulin, blood EBV-DNA, bone marrow invasion, and lymph node metastasis (*p* > 0.05). This study conducted a thorough analysis of the results and identified the following potential reasons for the negative results: firstly, the experimental group included only 40 cases, and a small sample size may have increased experimental error and impacted the results. Secondly, during the collection of clinical data, some data were missing, and patients did not undergo timely follow-up when their condition worsened, leading to delayed evaluation and adjustment of clinical staging. This, in turn, resulted in delayed updates of clinical data and increased experimental errors. In conclusion, we will consolidate our experience in future research, address these shortcomings, and aim for more reliable results.

The ROC curve, which is also referred to as the receiver operating characteristics curve, is a graphical representation with the true positive rate on the vertical axis and the false positive rate on the horizontal axis. It is commonly accepted that the closer the curve is to the upper- left corner, the more dependable the results become. By conducting ROC curve analysis, this investigation determined that the AUC values for both PLK1 and p-PLK1 did not exhibit significant differences. Nevertheless, owing to the limited number of cases in the experimental cohort and the abbreviated duration of follow-up, additional investigation is essential to ascertain the diagnostic potential of these markers in predicting the prognosis of NKTCL.

## Conclusions

In conclusion, the study demonstrated significantly elevated expression levels of PLK1 and its phosphorylation activity in NK/T-cell lymphoma compared to the control group. These findings suggest that PLK1 could serve as a potential diagnostic marker for NKTCL. Moreover, the positive correlation observed between the expression levels of PLK1 and p-PLK1 with the Ki-67 positive rate, indicates that PLK1 may influence cell proliferation, and their high expression is indicative of a poor prognosis. While this preliminary research highlights the potential role of PLK1 in the disease progression of NKTCL, the precise mechanism of abnormal PLK1 activation in NKTCL remains unclear. Therefore, further investigation is warranted to uncover the molecular mechanisms through which PLK1 promotes the onset and advancement of NKTCL. This will provide a theoretical foundation for exploring new treatment pathways for NKTCL.

## Data Availability

The authors confirm that the data supporting the findings of this study are available within the article.

## Abbreviations

NKTCL: NK/T cell lymphoma
PLK1: Polo-like kinase 1
p-PLK1: Phosphorylation of Polo-like kinase 1
WHO: World Health Organisation
LDH: Lactate dehydrogenase
OS: Overall survival
NHL: Non Hodgkin lymphoma
